# The barn owls’ Minimum Audible Angle

**DOI:** 10.1371/journal.pone.0220652

**Published:** 2019-08-23

**Authors:** Bianca Krumm, Georg M. Klump, Christine Köppl, Ulrike Langemann

**Affiliations:** 1 Cluster of Excellence “Hearing4all”, Division for Animal Physiology and Behaviour, School of Medicine and Health Sciences, Department of Neuroscience, Carl von Ossietzky University of Oldenburg, Oldenburg, Germany; 2 Cluster of Excellence “Hearing4all”, Division for Cochlea and auditory brainstem physiology, School of Medicine and Health Sciences, Department of Neuroscience, Carl von Ossietzky University of Oldenburg, Oldenburg, Germany; Universidad de Salamanca, SPAIN

## Abstract

Interaural time differences (ITD) and interaural level differences (ILD) are physical cues that enable the auditory system to pinpoint the position of a sound source in space. This ability is crucial for animal communication and predator-prey interactions. The barn owl has evolved an exceptional sense of hearing and shows abilities of sound localisation that outperform most other species. So far, behavioural studies in the barn owl often used reflexive responses to investigate aspects of sound localisation. Furthermore, they predominately probed the higher frequencies of the owl’s hearing range (> 3 kHz). In the present study we used a Go/NoGo paradigm to measure the barn owl’s behavioural sound localisation acuity (expressed as the Minimum Audible Angle, MAA) as a function of stimulus type (narrow-band noise centred at 500, 1000, 2000, 4000 and 8000 Hz, and broad-band noise) and sound source position. We found significant effects of both stimulus type and sound source position on the barn owls’ MAA. The MAA improved with increasing stimulus frequency, from 14° at 500 Hz to 6° at 8000 Hz. The smallest MAA of 4° was found for broadband noise stimuli. Comparing different sound source positions revealed smaller MAAs for frontal compared to lateral stimulus presentation, irrespective of stimulus type. These results are consistent with both the known variations in physical ITDs and variation in the width of neural ITD tuning curves with azimuth and frequency. Physical and neural characteristics combine to result in better spatial acuity for frontal compared to lateral sounds and reduced localisation acuity at lower frequencies.

## Introduction

Barn owls predominately hunt at night and rely on their auditory system to locate potential prey [1,2]. In situations like harsh winters or in the breeding season barn owls depend on their ability to strike prey for day-to-day survival [3]. In order to save energy, high localisation acuity is advantageous for hunting. Catching prey with high precision implies that the barn owl is capable of pinpointing the target with high precision [4–6]. In the present study we aimed to determine the barn owl’s behavioural sound localisation acuity in the laboratory.

Sound localisation in vertebrates largely relies on two binaural cues: interaural time (ITD) and interaural level difference (ILD). In mammals these cues are used for sound localisation in azimuth in a complementary fashion: ITDs are relied on to localize low-frequency sounds and ILDs to localize high-frequency sounds (referred to as duplex theory, see [7]). This means that binaural cues are analysed separately in low- and high-frequency processes [8,9]. Due to the structure of their fleshy pinnae, mammals are, in addition, able to use monaural spectral cues for sound localisation in elevation [9]. Similar to mammals, most birds use both ITD and ILD for sound localisation in azimuth. Barn owls, however, rely primarily on ITDs for sound localisation in azimuth, while ILDs play a minor role here [2,10–13]. Instead, the asymmetrical facial ruff of the barn owl generates ILDs that provide a cue for sound localisation in elevation [2,10,14].

Barn owls possess space-specific neurones that are tuned to specific sound source positions in azimuth and elevation. These neurones are located in the external part of the inferior colliculus (ICx) and represent a physiological map of auditory space [15]. Knudsen and his colleagues were the first to observe that space-specific neurones representing frontal space have more confined receptive fields, both in azimuth and elevation, than neurons tuned to locations in the periphery [15,16]. The azimuthal spatial tuning of neurones in the barn owl IC is determined mainly by the interaural time difference (ITD), corresponding to the large variation of this acoustic cue with azimuth [11]. As a general pattern, the width of the neurones’ ITD tuning curves increases with increasing azimuth and with decreasing frequency [17]. In other words, the ITD tuning is sharper for high- frequency sounds compared to low-frequency sounds and for azimuthal source positions in frontal space compared to lateral space. These observations predict that localisation acuity for sounds in the frontal space is superior compared to sounds presented in the periphery and that localisation acuity is also better for high-frequency compared to low-frequency sound sources. These assumptions are supported by behavioural studies in humans (*Homo sapiens*) [18], cats (*Felis silvestris catus*) [19] and European starlings (*Sturnus vulgaris*) [20].

Behavioural studies measuring the localisation of static sounds are classified either as absolute or relative localisation tasks [5,21]. An absolute sound localisation task requires the subject to identify the absolute position of a single sound source. Such a task measures localisation accuracy as well as localisation precision, corresponding to constant and random errors in sound localisation, respectively [4,5]. Absolute localisation tasks often evaluate motor actions like head or gaze orientation towards the perceived sound source position [10,22–25], and the localisation performance is often referred to as the minimum resolvable angle (MRA) [6]. In contrast, in a relative localisation task the position of one sound source is located with reference to another sound source. The thresholds are expressed as the minimum audible angle (MAA), a measure of localisation acuity [4,5,20]. A behavioural paradigm for deriving the MAA thus measures how well the position of a test stimulus can be discriminated from the position of a reference stimulus [18].

So far, behavioural studies measuring the barn owl’s localisation abilities have employed one of the following paradigms: pupillary dilation response (PDR) [26,27], head-turning behaviour [10,22] or target-approaching behaviour [1,28]. These studies used either reflexive responses, or trained the owls to fly to the target loudspeaker. Furthermore, they measured the localisation ability mainly in frontal space and predominately for the higher frequencies of the owls’ hearing range, i.e. above 3 kHz. In the present study, we determined the azimuthal MAA of barn owls using a Go/NoGo paradigm. We investigated the effects of stimulus type and the spatial position of the reference stimulus on the barn owls’ sound localisation acuity. We were able to compare the barn owls’ localisation acuity at high and low frequencies. Furthermore, we were able to compare frontal and lateral localisation acuity.

## Material and method

### Subjects

The subjects were 3 European barn owls (*Tyto alba*) aged between 2 and 24 years. Two of the birds hatched in 2015 at the University of Oldenburg, the third bird hatched in 1993 at the Technical University Munich. All birds were hand reared from the age of around 12 days. The owls were kept in individual indoor aviaries each and were transferred from the aviary to the experimental chamber on the fist. The food reward during the experiment and supplementary food consisted of pieces of one-day chickens (*Gallus gallus*). The owls' weight was monitored daily and the motivation of the animals was controlled by maintaining a weight of about 10 to 15% below their free feeding weight. The care and treatment of the birds were approved by the Landesamt für Verbraucherschutz und Lebensmittelsicherheit (LAVES), Lower Saxony, Germany (permit numbers: Az 33.9-42502-04-11/0647, Az 33.19-42502-04-16/2339).

### Experimental set-up

Experiments were carried out in a sound-attenuating echo-reduced chamber (IAC type 1203-A; outside dimensions: 2.8 m x 2.7 m x 2.5 m; internal dimensions: 2.2 m x 2.1 m x 2.0 m.), lined with sound-absorbing acoustic foam (PLANO 50/0 covered with WAFFLE 65/125 Seyboth & Co., cut-off frequency 500 Hz, α > 0.99; total attenuation 48 dB at 500 Hz, > 57 dB for frequencies ≥1 kHz). There was no cage, so the owls could move freely in the chamber. In the middle of the chamber was a pedestal to position the owl. Two perches were mounted on the pedestal, a waiting perch and a target perch. A stainless steel ring with a diameter of 1.8 m was wall-mounted 1.2 m above the floor. The ring supported 30 loudspeakers (Vifa XT25TG30-04, ASE) that were positioned at the height of the owl’s head. The loudspeakers were arranged in a semicircle with an azimuthal distance of 6° between two adjacent loudspeakers as viewed from the position of the head of the owl sitting on a waiting perch. To monitor the owl’s behaviour and location, the two perches were equipped with infrared light barriers. A custom-built automatic feeder was mounted in front of the target perch. The feeder provided up to 24 rewards for correct behavioural responses. A webcam (QuickCam Pro 9000, Logitech) was used to observe the owls during the experiments. A Linux-operated workstation controlled the experimental protocol by operating an enhanced real-time processor (RP2, Tucker-Davis Technologies). An external 32-channel soundcard (Hammerfall DSP Multiface II, RME) generated all acoustic stimuli. The soundcard's output was fed to four amplifiers (RMB-1048, Rotel) driving the 30 loudspeakers. Based on measured loudspeaker impulse responses, the sound pressure level and frequency responses of the 30 loudspeakers were equalized individually with a 128th order minimum phase FIR filter for each loudspeaker.

### Test signals

Stimulus types comprised narrowband noise signals (termed NB noise, bandwidth 40Hz) with centre frequencies of 500, 1000, 2000, 4000 and 8000 Hz and broadband noise signals (termed BB noise, frequency range 500 to 8000 Hz). For NB noise signal with a bandwidth of 40 Hz the transients and envelope fluctuations are slow [29]. Since all noise signals had the same bandwidths and did not provide distinct envelope cues, these do not provide useful ITDs for binaural comparison. Furthermore it has been shown that in the barn owl onset ITDs are unimportant in comparison to ingoing ITDs [30]. Signals had an effective duration of 100 ms (with 10 ms Hanning ramps) and were presented at a level of 40 dB SPL, which is well above the owls’ auditory threshold [31]. In order to rule out potential discrimination cues based either on loudness differences or on differences between single loudspeaker characteristics, a level roving of ±3 dB was implemented.

Stimuli were presented either from a single loudspeaker (true loudspeaker position), or from two adjacent loudspeakers simultaneously playing identical stimuli and thus simulating a sound source position between the two loudspeakers, using "summing localisation". This procedure was necessary in order to improve the spatial resolution of our setup. Due to their physical dimension the loudspeakers could not be moved any closer to each other. Previous studies had shown that this is a valid approach. Keller and Takahashi [32] have shown that an owl turns its gaze towards the space between the two loudspeakers when two speakers are activated simultaneously indicating that the owl perceived a single stimulus, located between two loudspeakers, i.e. the owl experienced summing localisation. Furthermore, Feinkohl and Klump [20] have shown that a starlings (*Sturnus vulgaris*) perception does not differ if stimuli are presented either from a "true" loud speaker position or from a position "simulated" by using summing localisation.

### Procedures

#### Go/NoGo task

The owls were trained in a Go/NoGo paradigm to report a change in stimulus position by leaving the waiting perch. The birds had learned to generally orient their head towards 0° in azimuth. A trial was started when the owl was sitting on the waiting perch and interrupting the light barrier. In each trial, reference stimuli were repeatedly presented within a random time interval of between 8 to 20 s ("waiting time"). Stimulus repetition rate was 1.3 s. When the waiting time had elapsed and the owl was still oriented towards 0° in azimuth, the next stimulus was presented from either another speaker location (test stimulus) or from the same speaker location (catch stimulus) as the reference stimulus. If the owl perceived the change in stimulus position it would fly to the target perch to obtain its food reward.

A response within a report interval of 5.5 s after the beginning of the test stimulus was scored as a “Hit”. Each “Hit” was rewarded with a piece of one-day chicken. In contrast, no response within the report interval was scored as a ‘‘Miss” and initiated the beginning of the next trial. In “catch trials” with no change of stimulus location the owl should remain on the waiting perch during the report interval. If the owl, however, would take off within the report interval in a catch trial, this was scored as a “false alarm” (FA). The false-alarm rate reflects a subject’s strategy and provides information about its decision criterion. If the owl would take off before the random time interval had elapsed (reference stimuli still running), this behaviour was rated as an “early alarm” (EA) and triggered a 10 s time out after which the waiting time restarted. Since test and catch stimuli are rare events, i.e., constitute only about 2% of the broadcast signals, the probability of random hits and false alarm is very low.

Thresholds were obtained by the method of constant stimuli [33], using a set of stimuli with a specific distribution of angular separations of the sound sources. These stimuli (test and catch stimuli) were presented in blocks of trials in random order. The number of blocks was different for the two paradigms (see below). In trials with test stimuli, the range of angular separation between reference and test was adjusted according to stimulus type and to the individual owl. Overall the angular separation varied between ±1.5° and ±27°. A negative sign denotes positions to the left of the reference whereas positive sign denotes positions to the right. Within one experimental session only one stimulus type (i.e. NB noise of a given centre frequency or BB noise) was tested. The direction of the change in speaker location, left or right from the reference position was randomized between trials. To minimize training effects, the sequence of stimulus types was randomized for each individual.

#### Single reference and multiple reference paradigms

We determined the minimum audible angle (MAA) with two different paradigms. In the single reference paradigm, the MAA was measured in frontal space (three owls, six stimulus types: NB noise centred at 500, 1000, 2000, 4000, 8000 Hz, BB noise). Here, the reference position was fixed at 0° in azimuth. Each session consisted of 42 trials divided into 6 blocks of 7 trials each. Each block consisted of 2 catch trials and a set of 5 test trials with different angular separations. In the multiple reference paradigm, the MAA was determined both in frontal and lateral space (two owls, four stimulus types: NB noise centred at 500, 2000, 8000 Hz, BB noise). The reference position varied between -45°, 0°, and +45° within one session. The reference position in each trial was selected in a pseudo-random order. Each session consisted of 42 trials divided into 2 blocks of 21 trials each. Each block consisted of 6 catch trials and a set of 15 test trials with five different angular separations for each of the three reference positions.

### Derivation of the minimum audible angle

To derive the owls' discrimination performance between reference and test stimuli, we employed the sensitivity measure d’ [34,35]. In the single reference paradigm four valid sessions were required to obtain the MAA. In the multiple reference paradigm ten valid sessions were necessary.

To obtain a psychometric function we first looked at the relationship between the proportion of correct responses and the angular separation between test and reference stimuli. Then we calculated the sensitivity for discriminating, separately for each angular separation. The thresholds defining the barn owls’ MAA were derived by fitting a cumulative normal distribution to the data points constituting the psychometric function and by estimating the angular separation at which a d’ of 1.0 was reached (see S1 Fig for an example).

In the single reference paradigm, a session was considered valid if (1) the owl achieved at least 12 Hits, (2) the false alarm rate was ≤ 20%, (3) the d’ value of the smallest angular separation was below 1.0, (4) the d’ value exceeded 1.8 for one of the larger angular separations. Since it became apparent that the first two validity criteria were already good predictors for a stimulus driven response, the number of validity criteria were reduced for the second paradigm. Thus in the multiple reference paradigm, experimental sessions were included in the analysis if (1) the owl achieved at least 12 Hits and (2) the false alarm rate was ≤ 20%.

### Data analysis

We performed a general linear mixed model (GLMM) analysis of variance to test for the effects of stimulus type and reference position. The dependent variable was the MAA. Statistical analyses were performed using SPSS 24.0 (IBM SPSS Statistics). A p value of ≤ 0.05 was the criterion for a significant effect. Since there was no significant difference between the frontal MAAs obtained with either paradigm, the data of the single reference and the multiple reference paradigms were pooled in the analysis.

## Results

We determined the barn owl’s relative localisation acuity, i.e., the minimum audible angle. The owls’ MAA was strongly affected by the factors stimulus type and reference position. There was no significant difference between the individual owls.

### Effect of stimulus type on localisation acuity

**Fig 1:**
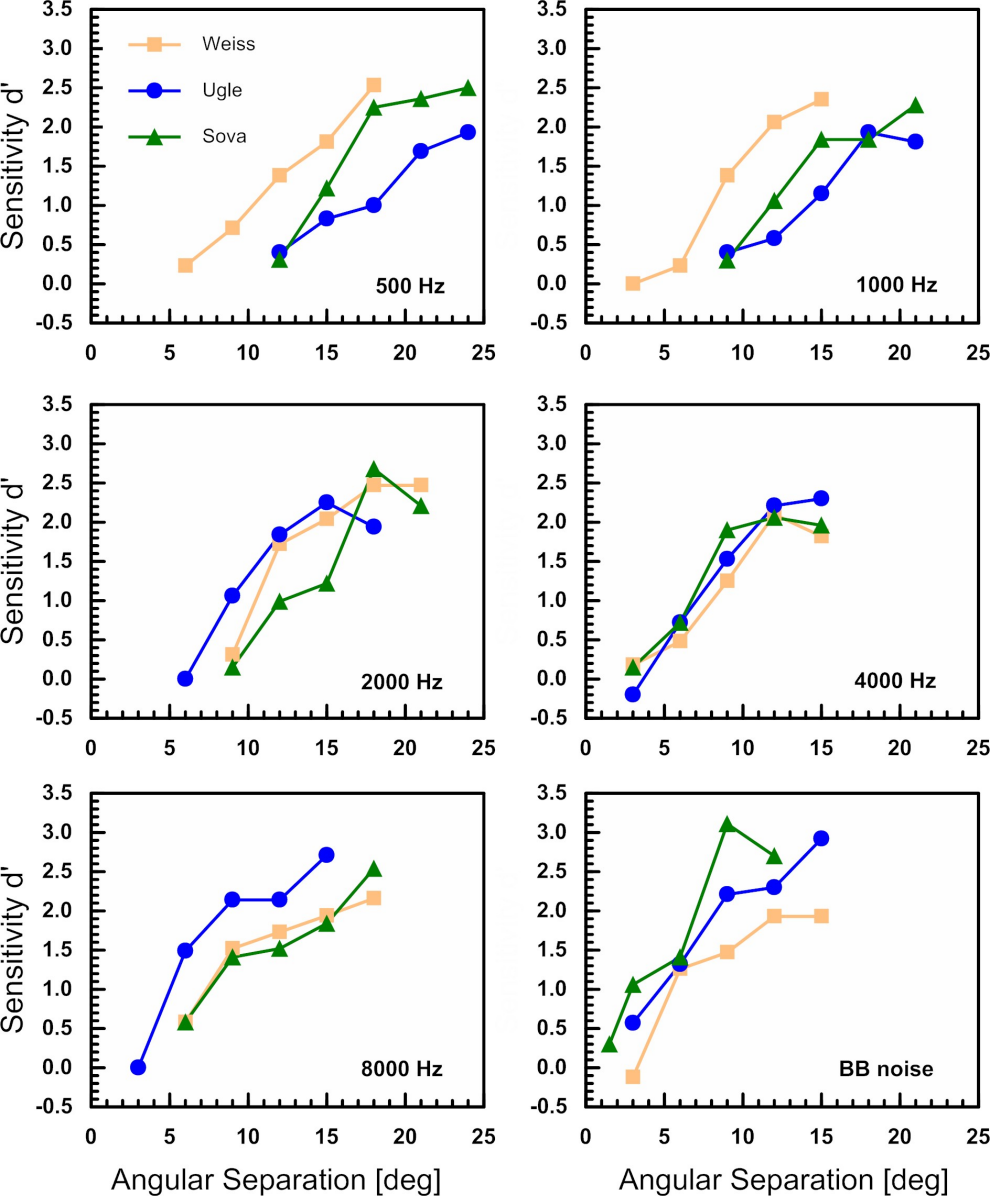
Psychometric functions for all six stimulus types and three owls obtained with a single reference position at 0° in azimuth. The barn owls sensitivity (d’) is plotted as a function of angular separation between reference and test stimuli. The different colours represent the different owls.

The barn owls’ individual psychometric functions observed in the single reference paradigm are depicted in Fig 1. These functions show that the sensitivity varied between a d’ of about 0 and 3. Furthermore, the steepness of the psychometric functions increased with increasing test frequency. Individual MAA values that were obtained in either paradigm are listed in Tables 1 and 2. Generally the owls’ MAA decreased with increasing centre frequency. In the single reference paradigm, the largest MAA of 13.7° ± 3.2° (mean ± s.d.) was obtained at 500 Hz and MAA decreased to 8.4° ± 1.4° at 8000 Hz (Table 1). With BB noise, the MAA further decreased to 4.9° ± 0.3°. The owls’ localisation acuity differed significantly between stimulus types (F = 39.942, p < 0.001). Pairwise comparison (Bonferroni corrected) revealed that localisation acuity for all stimulus types was significantly different from that for the 500 Hz condition (p < 0.001). In addition, acuity obtained with BB noise was significantly different to that for the 1000 Hz, 2000 Hz and 8000 Hz conditions (p = 0.014, p < 0.001, and p = 0.007, respectively). Furthermore, localisation acuity for the 4000 Hz condition was significantly different from that for the 1000 Hz and the 2000 Hz conditions (p = 0.047 and p < 0.001, respectively). All other comparisons yielded no significant results.

**Table 1:** MAA values in degree, mean values and s.d. of three barn owls (Ugle, Sova, and Weiss) as a function of stimulus type. Data were obtained in the single reference paradigm.

stimulus type	Ugle	Sova	Weiss	mean	s.d.
**500 Hz**	15.0	16.0	10.0	13.7	3.2
**1000 Hz**	12.3	12.9	7.7	11.0	2.8
**2000 Hz**	9.8	13.1	12.3	11.7	1.7
**4000 Hz**	7.3	6.6	6.3	6.7	0.5
**8000 Hz**	7.0	9.8	8.5	8.4	1.4
**BB noise**	4.9	4.6	5.2	4.9	0.3

**Table 2:** MAA values in degree of two barn owls (Ugle and Sova) as a function of reference position and stimulus type. Data were obtained in the multiple reference paradigm.

reference position	stimulus type	Ugle	Sova
**- 45°**	500 Hz	20.3	19.7
	2000 Hz	13.8	12.9
	8000 Hz	10.9	11.7
	BB noise	8.8	7.2
**0°**	500 Hz	14.1	14.9
	2000 Hz	10.2	10.1
	8000 Hz	5.5	7.7
	BB noise	4.1	2.7
**+ 45°**	500 Hz	20.0	19.7
	2000 Hz	13.5	13.0
	8000 Hz	10.7	12.5
	BB noise	10.2	6.6

### Effect of reference position on localisation acuity

**Fig 2:**
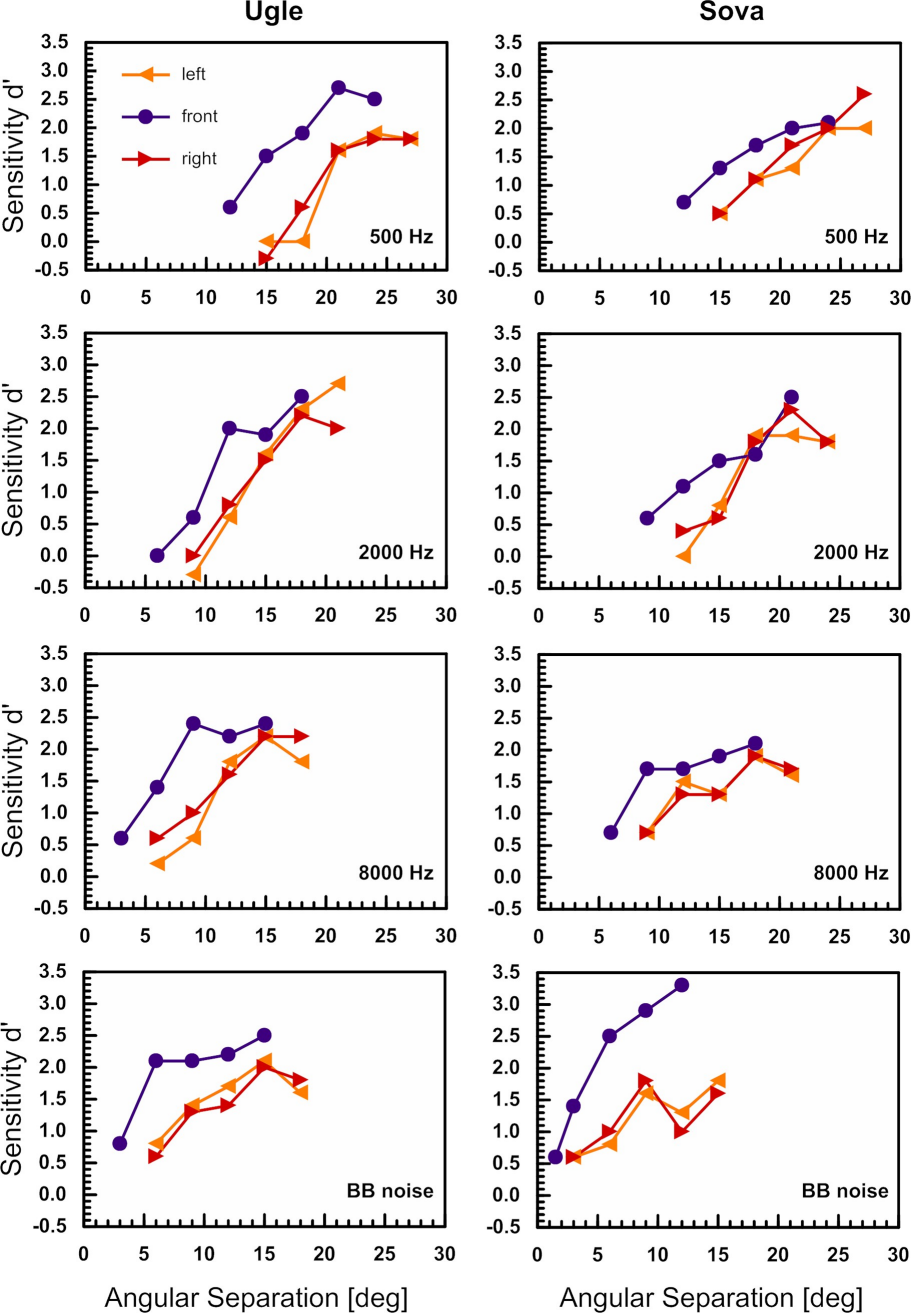
Psychometric functions for four stimulus types and two owls obtained in the multiple reference paradigm (reference stimuli were presented from -45°, 0°, and +45°). The barn owls sensitivity (d’) is plotted as a function of angular separation between reference and test stimuli. The different colours represent the different reference positions.

In the multiple reference paradigm, reference stimuli were presented from three azimuthal positions (-45°, 0°, +45°). The psychometric functions of two barn owls, obtained in the multiple reference paradigm, are shown in Fig 2. The psychometric functions of the lateral reference positions were both shifted to the right relative to the frontal reference. This corresponds to a larger MAA for lateral compared to frontal stimulus presentation. Irrespective of the stimulus type, the “frontal MAA” was significantly smaller compared to the “lateral MAA” (see statistical results below). For any of the three reference positions, MAA values increased with decreasing centre frequency, similar to the data obtained with frontal stimulus presentation only (Fig 1). Again, the largest MAA values were obtained at 500 Hz (Table 2). “Lateral MAAs” were on average 19.9° at 500 Hz, 13.3° at 2000 Hz, 11.5° at 8000 Hz, and 8.2° for BB noise. “Frontal MAAs” obtained in the multiple reference paradigm were on average 14.5°, 10.2°, 6.6°, 3.4°, at 500 Hz, 2000 Hz, 8000 Hz and BB noise, respectively (Table 2). The barn owls’ localisation acuity differed significantly between reference positions (F = 26.829, p < 0.001). Pairwise comparisons (Bonferroni corrected) revealed a significant difference between the frontal and each of the lateral reference positions (p < 0.001), but no significant difference between the two lateral reference positions. For the lateral positions we tested whether the direction of the change in stimulus position had a significant effect on the barn owls' behaviour, i.e., we checked whether the behavioural response was biased towards "inward" or "outward" shift of location (relative to the reference position). We did not find such an effect (chi-square tests, using Hit and Miss responses). Moreover, there was no interaction between stimulus type and reference position indicating that stimulus types and reference position had an additive effect. In other words, the change of MAA with centre frequency was similar at all reference positions and the MAA was generally smaller in frontal space compared to lateral space.

## Discussion

In this study, we investigated the barn owls’ MAA for different stimulus types and different spatial locations. The different stimulus types covered nearly the entire hearing range of the barn owl [31,36]. In particular, we strove to also cover the low-frequency range (< 3 kHz) which had only been sampled sparsely in previous studies [10], allowing for a valid comparison of localisation acuity at high and low frequencies. Furthermore, using a conditioned behavioural response, different reference positions could be tested, enabling for the first time the comparison between frontal and lateral localisation acuity.

### Stimulus type and localisation acuity

**Fig 3:**
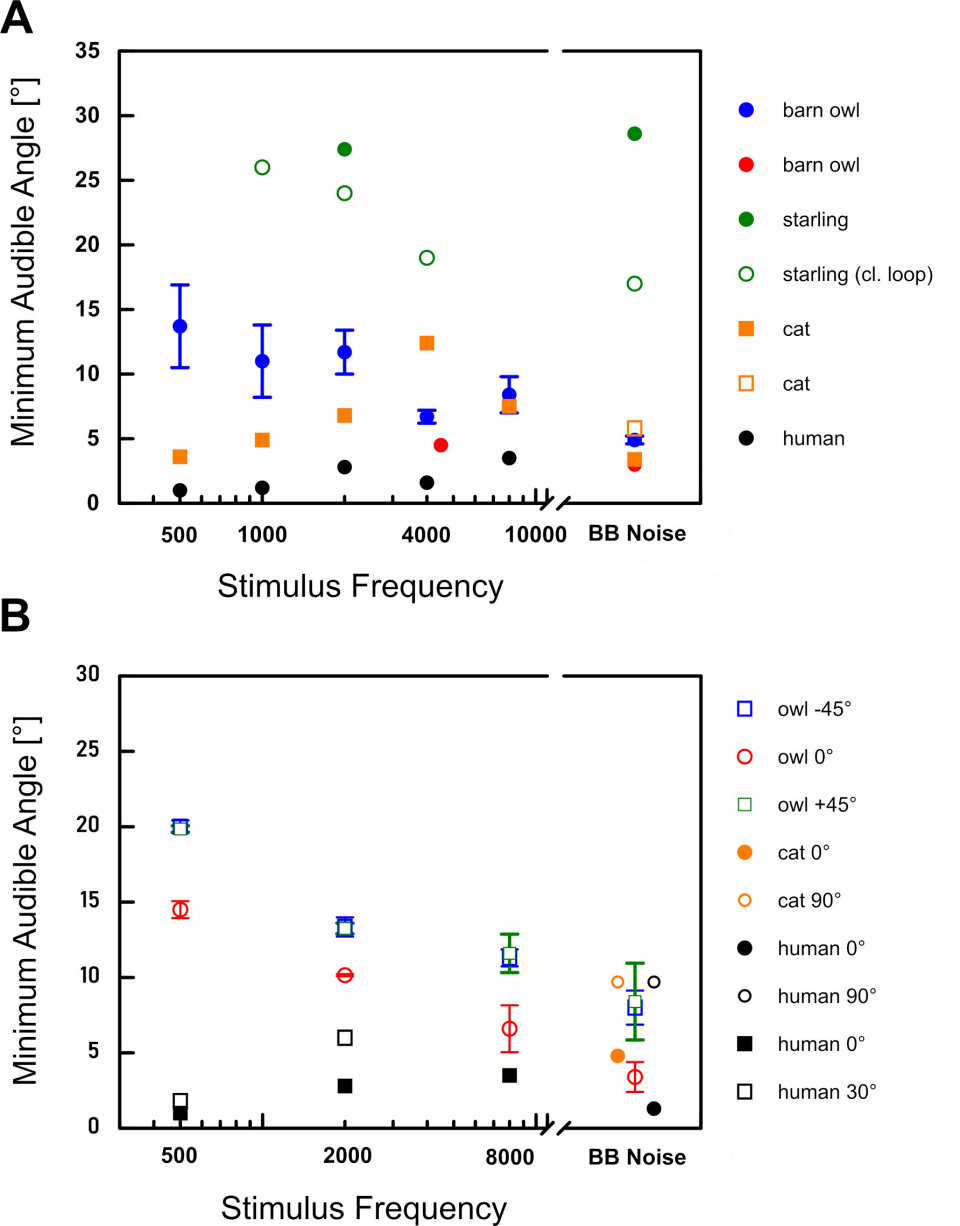
Minimum Audible Angle (MAA) as a function of stimulus type in different species. Data from different sources: **(A)** owl (blue dot, present study, N = 3), owl (red dot, Bala and Takahashi 2000, N = 5), starling (green dot, green circle, Feinkohl and Klump 2013, N = 4), cat (orange squares, Martin and Webster 1987, N = 5), human (black dot, Mills 1958, N = 3); **(B)** owl (blue square, red circle, green square, present study, N = 2), cat (orange dot, orange circle, Heffner and Heffner 1988, N = 4), human (black dot, black circle, Heffner and Heffner 1988, N = 4), human (black squares, Mills 1958, N = 3). The reference position in **(A)** was at 0° in azimuth and in **(B)** different reference positions were used.

Under natural conditions, barn owls will hear prey signals that are more likely broadband than narrowband in frequency, for example, rustling leaves stirred up by a mouse rushing to cover [1]. Broadband signals will stimulate a considerable proportion of the barn owl’s sensory epithelium in the inner ear and thus provide multiple localisation cues in a larger frequency range than narrowband signals. Hence sound localisation acuity for broadband signals is expected to be superior to localisation acuity for narrowband signals. The results from our study confirm that the barn owls’ localisation acuity is indeed best with BB noise. The MAA for BB noise signals presented within frontal space was about 4°. This remarkable acuity is in accordance with previous studies [10,22,27] investigating the sound localisation ability of the barn owl. Similar to our study, the behavioural studies referred to in Table 3 used either broadband and narrowband stimuli or tones. Typically, the behavioural localisation performance deteriorates with decreasing centre frequencies. This was also true for the barn owls’ MAA values obtained in our study (Fig 1, Table 1, and Fig 3A). A study by Cazettes et al. [17] indicates that the width of ITD tuning curves of space-specific neurones in the external part of the inferior colliculus (ICx) becomes narrower with increasing frequency (Fig 4A in [17]). Since neural ITD responses are based on measurements of interaural phase difference (IPD), this is a direct consequence of the IPD sensitivity of their input neurones that are narrowly frequency selective [37]. In particular, the period length of a stimulus becomes shorter as the frequency increases [37], which also requires a higher temporal precision of the neurons [38].

**Table 3:** Barn owl sound localisation performance for different stimulus types measured in selective studies.

Study	N	procedure	threshold criterion	Stimuli	localisation ability [°]
**Knudsen and Konishi 1979**	1	Search coil technique	head turning accuracy (localisation error)	2 kHz 75 ms 3 kHz 75 ms 4 kHz 75 ms 6 kHz 75 ms 8 kHz 75 ms 9 kHz 75 ms 10 kHz 75 ms BB noise 75 ms	12 11 10 11 12 11 20 5
**Knudsen et al. 1979**	1	Search coil technique	head turning accuracy (localisation error)	BB noise 75 ms	3
**Bala and Takahashi 2000**	3	MAA, Pupillary dilation response (PDR)	D > 0.8	4.5 kHz 100 ms BB noise 100 ms	5 3
**Bala et al. 2003**	3	MAA, Pupillary dilation response (PDR)	D > 0.8	BB noise 100 ms	3
**Present study**	3	MAA, Go/NoGo	d’ 1.0 (equivalent to 0.76 p(c))	0.5 kHz 100 ms 1 kHz 100 ms 2 kHz 100 ms 4 kHz 100 ms 8 kHz 100 ms BB noise 100 ms	14 (± 3.5) 11 (± 3.1) 11 (± 1.7) 7 (± 0.7) 7 (± 1.4) 4 (± 1.3)

Although the behavioural sensitivity of barn owls extends to frequencies as low as about 200 Hz [36], and even lower best-frequency responses have been observed neurally [39], no previous behavioural sound localisation study has used stimuli with an centre frequency lower than 1000 Hz (Table 3). In our study, we determined the barn owl’s localisation acuity down to a centre frequency of 500 Hz. The barn owl’s MAA was about 14° at 500 Hz, a value that is nearly twice as large as the MAA of 8° obtained at 8000 Hz. In contrast, estimates of human sound localisation acuity at 500 and 8000 Hz revealed an MAA of about 1° and 3.5°, respectively [18] (Fig 3A). At first glance, human subjects thus appear to outperform barn owls. Humans, however, benefit from a much larger head that provides larger ITD and ILD cues. Generally, MAA values obtained in humans are smallest in the low-frequency range which plays a major role in communication [18,40]. A more appropriate comparison to owls with respect to head size is the cat, a predatory mammal with good low-frequency hearing and a slightly larger head than the barn owl (cat about 60 mm, barn owl about 44 mm) [41–43]. Martin and Webster [44] showed that the cat’s MAA for BB noise was about 4°. The cat’s MAA values for pure tones generally increased from 0.5 to 32 kHz [44]. This is in contrast to the barn owl, were MAA values generally decrease with increasing frequency. One explanation for that is, that the cats’ auditory system shows phase locking (the temporal synchrony to the waveform) only up about 4 kHz [45]. Thus ongoing ITDs can only be neurally represented and used by the cat at frequencies below 4 kHz. At higher frequencies, ILDs become more effective [44,45].

Barn owls are most sensitive at frequencies between about 4 and 8 kHz [36], rely mainly on high-frequency cues for hunting [2] and show their smallest MAA at 8 kHz. Since the barn owl’s auditory system shows phase locking up to 10 kHz, barn owls are able to use ITDs at high frequencies [38,46]. Furthermore high frequencies are over-represented on the barn owl’s basilar papilla: more than half of its length represents the frequency range between 4 and 8 kHz [47]. Based on all these findings, head size alone clearly is an unsuitable predictor for sound localisation acuity (see also [48]). Instead, ecological constraints like catching prey or sensitivity for communication signals might put sound localisation abilities and the underlying neural mechanisms under more or less selective pressure in different species [39].

Previous behavioural studies in the barn owl have also used different modes of stimulus presentation. Stimuli were either presented in a closed-loop condition (allowing for head turns during the presentation of the sound) [10,22,28] or in an open-loop condition (stimuli terminate before the subject can initiate head turns in response to the sound) [10,22]. Bala and colleagues [26,27] presented their stimuli also under an open-loop condition, however, instead of orienting responses they measured pupillary dilation responses with the head fixated. It has been previously shown that stimulus duration affects localisation performance [49]. Stimulus duration can either support open-loop or closed-loop behavioural responses [21]. Closed-loop experiments with long stimulus durations allow the subjects to correct the initial trajectory of the behavioural responses based on the sensory feedback [21]. Therefore, long stimulus durations (closed-loop condition) can yield better sound localisation thresholds (e.g. lower MAA or MRA values) than short stimulus durations [20,50]. In the starling it has been shown that the mean MAA for BB noise improved by 11.2° when stimulus duration was increased from 100 ms to 1000 ms, whereas the mean MAA for a 2 kHz tone improved by 3.2° when presented with a long stimulus durations of 1000 ms [20]. Since closed-loop experiments do not provide much information about the mechanism of sound localisation, it is difficult to draw valid conclusions on the subjects’ localisation acuity. Thus, data obtained under closed-loop conditions are not directly comparable to data obtained under open-loop conditions. Table 3 therefore refers only to behavioural studies that presented stimuli under open-loop conditions.

### Comparison of localisation acuity with physical cues

Azimuthal sound localisation acuity in the barn owl depends on the ability to process differences in the arrival times of sounds at the two ears (ITDs). Sensitivity to ITDs depends on temporal coherence of the timing of the action potentials and the waveform (i.e., phase locking). The temporal information of a stimulus is encoded by phase locking of auditory-nerve fibre responses [51]. Avian auditory-nerve fibres typically phase lock up to 3–4 kHz [38]. The barn owl, however, extends phase locking up to frequencies of about 10 kHz [38,46]. The quality of phase locking is commonly expressed as vector strength. It is known that vector strength decreases with increasing frequency [38]. In the case of the barn owl, the vector strength of phase locking decreases from 0.9 at 0.4–0.5 kHz to 0.2 at 9 kHz [38].

Head-related transfer functions (HRTFs) are direction-specific acoustic filters that represent all relevant cues that might be used by the auditory system for locating sound sources in space. It is possible to derive single spatial cues from HRTFs. We used HRTFs of barn owls [52] to convert our MAA values (depicted in Tables 1 and 2) into the corresponding ITDs. We estimated the change in ITD per degree in azimuth using the slope of a regression line (panel B of S2 Fig). The slope of the ITD representing frontal space, within ±10° in azimuth, was about 5 μs/degree for 0.5 kHz and only about 2.5 to 4 μs/degree for frequencies of 1 kHz and above. Using these slopes we derived “minimum audible ITDs” of about 71 μs, 44 μs, 44 μs, 27 μs, and 19 μs, for 500, 1000, 2000, 4000 and 8000 Hz, respectively. The slope of the ITD representing between ±35° and ±55° lateral azimuth was about 3.5 μs/degree for 0.5 kHz and about 1.4 to 4 μs/degree for frequencies of 1 kHz and above. Using these slopes, we derived minimum audible ITDs of about 70 μs, 33 μs, and 46 μs, for 500, 2000, and 8000 Hz, respectively. Note that since the frequencies of HRTF and behavioural did not correspond exactly, we matched 3000 and 7000 Hz from HRTF data to MAAs at 4000 and 8000 Hz, respectively.

HRTF recordings, however, do not consider the effect of the internally coupled middle ears of birds [53]. The interaural connections are assumed to amplify sound localisation cues, like the ITD, especially in the low frequency [51,53]. According to that, HRTFs do not reflect what the barn owl actually perceives, especially at low frequencies. To reevaluate the effect of the internally coupled ears, Kettler and colleagues [54] measured eardrum vibrations in the barn owl, by using laser Doppler vibrometry. It was shown that low-frequency directionality increased with decreasing frequency in a narrow frequency band from 1.5 and 3.5 kHz [54]. Even though an effect of the internally coupled ears on directionality was apparent below 1.5 kHz, the authors stated that their eardrum vibration measurements were only reliable in a frequency range between 1.5 and 6.3 kHz [54].

More data on the directionality of the peripheral auditory system responses at low frequencies and on the contribution of the interaural connections are provided by a study from Calford and Piddington [55]. They measured cochlear microphonics (CM) in the grass owl (*Tyto longimembris*), a *Tyto* species with a head size similar to that of the barn owl (*T*. *longimembris* 42.5 mm [55]; *T*.*alba* about 45 mm [42]). The results of Calford and Piddington [55] demonstrated that the overall ITD range available to the owl is clearly frequency dependent. The ITD range at frequencies above 1 kHz was similar to an ITD range estimated mainly by the path length around the head (approx. ±150 – ±220 μs) and as shown in HRTFs (S2 Fig). In contrast, at frequencies below 1 kHz, the ITD range available to the owl was much larger, up to ±400 and ±550 μs [55]. This supports the hypothesis that the internal coupling of the middle ears has a greater role at lower frequencies whereas at higher frequencies, these interaural connections appear to be functionally irrelevant [55,56] (S2 Fig). At higher frequencies, the ITD range perceived by the owl should thus approximate its acoustic range of ITDs (measured as ±250 to ±300 μs [52], S2 Fig).

From the CM-derived relation between sound source azimuth and ITD in the grass owl [55] we estimated the change in ITD per degree in azimuth using the slope of a regression line (panel B of S2 Fig). The slope of the ITD representing frontal space, within ±10° in azimuth, was about 11 μs/degree for 0.5 kHz but only about 2 to 5 μs/degree for frequencies of 1 kHz and above. The steeper slopes for frequencies below 1 kHz clearly reflect the contribution of the internally coupled middle ears, i.e., ITDs perceived by the owl are enhanced (which should improve localisation acuity at low frequencies). For the barn owls’ MAAs, these slopes predict the following equivalent minimum audible ITDs: the frontal MAA values for 500, 1000, 2000, 4000 and 8000 Hz correspond to an ITD of about 155 μs, 50 μs, 55 μs, 20 μs and 15 μs, respectively. The slope of the ITD representation between ±35° and ±55° lateral azimuth was about 4 μs/degree for 0.5 kHz and about 2 μs/degree for frequencies of 1 kHz and above. The lateral MAA values for 500, 2000 and 8000 Hz thus correspond to minimum audible ITDs of about 80 μs, 30 μs and 23 μs, respectively. These numbers for minimum audible ITDs are clearly larger for frequencies below 1 kHz than those for frequencies above 1 kHz. In addition, for 2000 Hz and 8000Hz, frontal and lateral minimum audible ITDs are similar while for 500 Hz, frontal minimum audible ITDs are considerably larger than lateral ones.

Note that the above conversion of behavioural MAA to minimum audible ITD normalises out all known variations of the physical ITD cue, whether they arise from differential diffraction in frontal vs. lateral space, or from the internally coupled middle ears across frequencies. In other words, if the neural processing of ITD were invariant across frequencies, the minimum audible ITD should not vary across frequency. However, it clearly does. This indicates that the underlying neural computation of ITDs is, firstly, frequency dependent (more accurate at high than at low frequencies) and secondly, for a frequency of 500 Hz, is more accurate for lateral sound source positions than for frontal ones. The fact that the neural computation of ITDs is less accurate at lower frequencies might be explained by the temporal dispersion in phase locking. Even though the quality (vector strength) of phase locking decreases with increasing frequency, the temporal dispersion (temporal jitter) is reduced because the period of the sound signal decreases with increasing frequency [37,57,58]. This results in an enhanced temporal precision at higher frequencies compared to low frequencies [58].

### Localisation acuity as a function of sound source position

In the present study, the barn owls’ localisation acuity was best when the reference stimuli were presented from 0° in azimuth and the test stimuli were also presented within frontal space. Irrespective of stimulus type, MAA values deteriorated when the reference stimulus was shifted into lateral space (Fig 3B). This is in accordance with a previous study in the barn owl using head-turning responses as a measure of localisation accuracy in an absolute localisation task [22]. Here the localisation error for a target sound presented within ± 10° in azimuth was about 2° (frontal space, open-loop condition, BB noise). The localisation error increased to about 6° and 9° for target sounds presented at ± 50° and ± 70°, respectively [22]. When measuring azimuthal head-turning behaviour barn owls generally tended to underestimate the position of a noise burst presented laterally, i.e. their localisation accuracy decreased [10,59]. This implies a bias towards frontal sound source positions. The barn owl’s higher sound localisation acuity in frontal space not only holds for stationary sounds. They are also more sensitive in distinguishing leftward and rightward moving auditory stimuli presented in frontal space compared to lateral space [60].

Reduced localisation acuity for lateral sounds was also observed for behavioural responses of cats and humans (Fig 3B). Mills [18] showed for human subjects that localisation acuity of tones was better in front compared to the periphery. The smallest MAA values for humans were about 1° at frequencies between 250 and 1000 Hz for a reference position of 0° in azimuth. The MAA increased when reference positions were shifted into lateral space. For frequencies below 1000 Hz, the MAA values were about 2.5 and 7° for reference positions of 45° and 75°, respectively [18]. At higher frequencies, MAA values were much larger (see [18]. Heffner and Heffner [19] obtained similar results in humans and cats with broadband noise. For humans they reported thresholds of 1.3°, 2.8°, 4.4° and 9.7° for sound sources centred at 0°, 30°, 60°, and 90°, respectively [19]. In cats the thresholds for broadband noise signals increased from 4.8° to 7.5°, 6.8°, and 9° for 0°, 30°, 60° and 90°, respectively [19]. In an absolute localisation task Nodal and colleagues [24] found that the ferret's (*Mustela putorius furo*) localisation performance also decreased when test signals were presented from lateral positions [24]. All of these behavioural studies show best localisation performance in frontal space, irrespective of head sizes and ecological constraints. Since azimuthal sound localisation in mammals depends both on ITDs and ILDs, localisation acuity might not only be affected by ITDs. ILDs have been shown to become non-monotonic with increasing azimuth and thus ILDs possibly effect the localisation performance (e.g. [61]). In contrast to mammals, barn owls exploit ITD and ILD cues differently. CM recordings as well as HRTF recordings in the barn owl have shown that ITDs vary almost exclusively with azimuth, while ILDs vary less in azimuth than in elevation and are thus predicted to play only a minor role in azimuthal sound localisation [11,14,62]. Indeed, head orienting responses to dichotic stimuli delivered via headphones confirmed that ILDs vary only slightly with azimuth [12]. From these results we conclude that the barn owls’ localisation acuity probably was mainly affected by ITD cues and less by ILD cues.

In general, neurophysiological studies in the barn owl midbrain also suggested that localisation acuity should be better in frontal space compared to the periphery. Knudsen and colleagues found that the majority of space-specific neurones were tuned to the frontal space (about ± 30° in azimuth) and had more confined receptive fields in both azimuth and elevation than neurones tuned to lateral space [15,16]. Thus, the frontal space is clearly overrepresented in the barn owls’ neural map of auditory space [15,16]. In addition, Cazettes and colleagues [17] conclude that the ITD tuning width of the space-specific neurones in the ICx varied as a function of azimuthal location [17]. Fischer and Peña [63] applied a model to explain the barn owls’ better performance at frontal positions, i.e., superior localisation performance in frontal space than in lateral space. By using a Bayesian estimator, they were able to explain the sound localisation performance. Our behavioural observations on the MAA and the corresponding minimum audible ITDs for different frequencies (see above) are in agreement with these neurophysiological results.

Interestingly, there also is a match between physiology and behaviour of the barn owl when relating the minimum audible ITDs derived from the MAA and frequency-specific variation in spatial tuning of ICx neurones. Cazettes and colleagues [17] observed that high-frequency neurones in the ICx had more narrow ITD tuning curves than low-frequency neurones. Furthermore, neurons tuned to low frequencies preferentially responded to larger ITDs (corresponding to lateral sound source positions) whereas neurones tuned to higher frequencies preferentially responded to smaller ITDs, i.e., frontal sound source positions [64]. This matched the physical cue reliability of interaural phase differences for natural stimuli filtered by a typical owl’s HRTF: low frequencies provide a more reliable cue for stimuli emanating in lateral space and vice versa, i.e. better reliability of high-frequency components for stimuli emanating in frontal space [17,64]. Finally, Cazettes et al. [65] showed that these biases are conveyed, via convergent projections, to pre-motor neurones driving the owl’s spontaneous head-turning behaviour. Mean firing of the pre-motor neurone population predicted the owl’s general behavioural bias for greater localization accuracy in frontal vs. lateral space and also its greater bias when responding to high-frequency as compared to low-frequency band-pass noises [65].

For the owl’s behaviour in the present experiments, this predicts that the minimum audible ITDs corresponding to frontal and lateral MAA values are frequency-dependent. As shown above, the derived values for the minimum audible ITDs corresponding to frontal and lateral MAAs are consistent with these predictions. As expected based on the ITD tuning of ICx neurones at different frequencies, minimum audible ITDs were smaller at high frequencies than at low frequencies. Furthermore, whereas for high frequencies the minimum audible ITD was larger for lateral than for frontal positions, at the lowest frequency of 500 Hz the minimum audible ITD was the same for lateral and frontal positions. This is in agreement with the observation of a greater bias towards frontal space in the accuracy of spontaneous head turns to high-frequency band-pass noises when compared to those elicited by low-frequency band-pass noises [65].

## Supporting information

S1 Fig Description of fitting procedure for estimating the minimum audible angle (MAA). The data points representing response rate in relation to the angular separation of the sound sources were fitted by a cumulative normal distribution, applying the Generalized Reduced Gradient nonlinear method for minimizing the total RMS error of the differences between the fitted values and the measured data points using the "Solver Add-in" in the Microsoft Excel version 2010. Adjusted parameters of this fitted psychometric function (blue line) were the lapsing rate (i.e., upper limit), the false-alarm rate (i.e., lower limit), the inflection point and the slope of the function at the inflection point. Based on the false-alarm rate, the hit rate that represented a sensitivity d’ of 1.0 for detecting the change in sound source location was calculated. For a false-alarm rate of 0.071 and a threshold d’ of 1.0 the hit rate at threshold is 0.319. Based on this threshold hit rate (green arrow) the threshold angular separation (black arrow, i.e., the MAA) of 4.9° is determined from the fitted psychometric function.
(TIF)

S2 Fig ITD as a function of frequency and azimuth. **(A)** Dependence of ITD was calculated from cochlear microphonics (CM; closed symbols and solid lines) and head-related transfer functions (HRTF; open symbols and dashed lines). Data are estimated from Moiseff 1989 [11] (*CM Barn owl*, *N = 1*), from Calford and Piddington 1988 [55] (*CM Grass owl*, *N = 1*), and from Hausmann et al. 2010 [52] (*HRTF Barn owl*, *N = 1*). HRTF data represent the passive acoustic case, and CM data represent the internally coupled ears case. (**B**) From the data depicted in (A) we calculated the slopes of the ITD representation. Slopes representing frontal space within ±10° in azimuth are represented by closed symbols and solid lines, whereas slopes representing lateral space are represented by open symbols and dashed lines. The calculation of the lateral slopes are based on data points that approximately correspond to our lateral reference positions of ±45°. For the *CM Grass owl* data and the *HRTF Barn owl* data we considered data points within ±35° to ±55° in azimuth, and for the *CM Barn owl* data we could only consider data points within ±30° to ±50° in azimuth.
(TIF)
