# Supplementary figures and images for: The barn owls’ Minimum Audible Angle
Source: PLoS One. 2019 Aug 23;14(8):e0220652. doi: 10.1371/journal.pone.0220652 (PMC6707599; doi:10.1371/journal.pone.0220652)

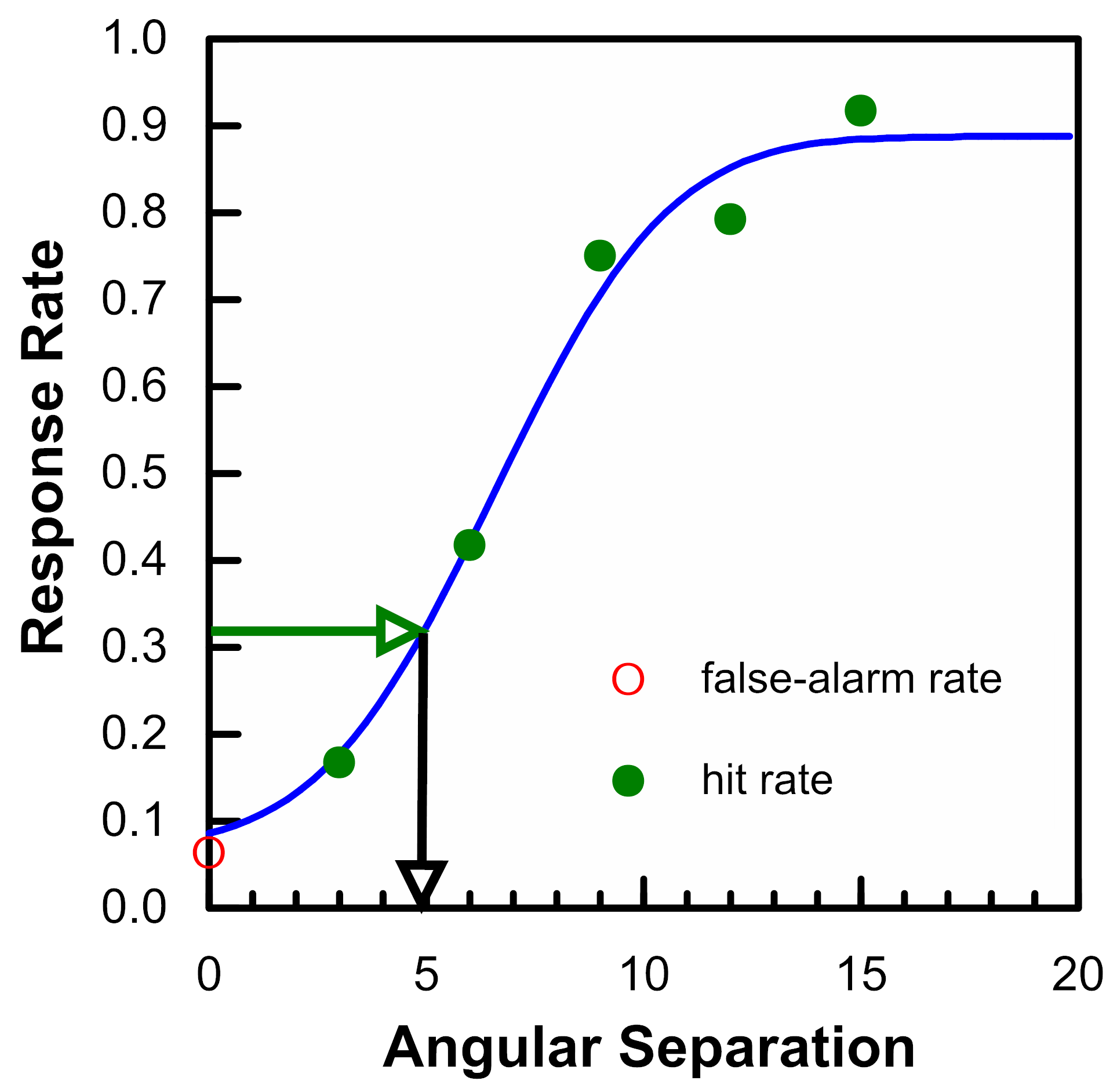

Supplement: S1 Fig — The data points representing response rate in relation to the angular separation of the sound sources were fitted by a cumulative normal distribution, applying the Generalized Reduced Gradient nonlinear method for minimizing the total RMS error of the differences between the fitted values and the measured data points using the "Solver Add-in" in the Microsoft Excel version 2010. Adjusted parameters of this fitted psychometric function (blue line) were the lapsing rate (i.e., upper limit), the false-alarm rate (i.e., lower limit), the inflection point and the slope of the function at the inflection point. Based on the false-alarm rate, the hit rate that represented a sensitivity d’ of 1.0 for detecting the change in sound source location was calculated. For a false-alarm rate of 0.071 and a threshold d’ of 1.0 the hit rate at threshold is 0.319. Based on this threshold hit rate (green arrow) the threshold angular separation (black arrow, i.e., the MAA) of 4.9° is determined from the fitted psychometric function. (TIF) [file pone.0220652.s001.tif]

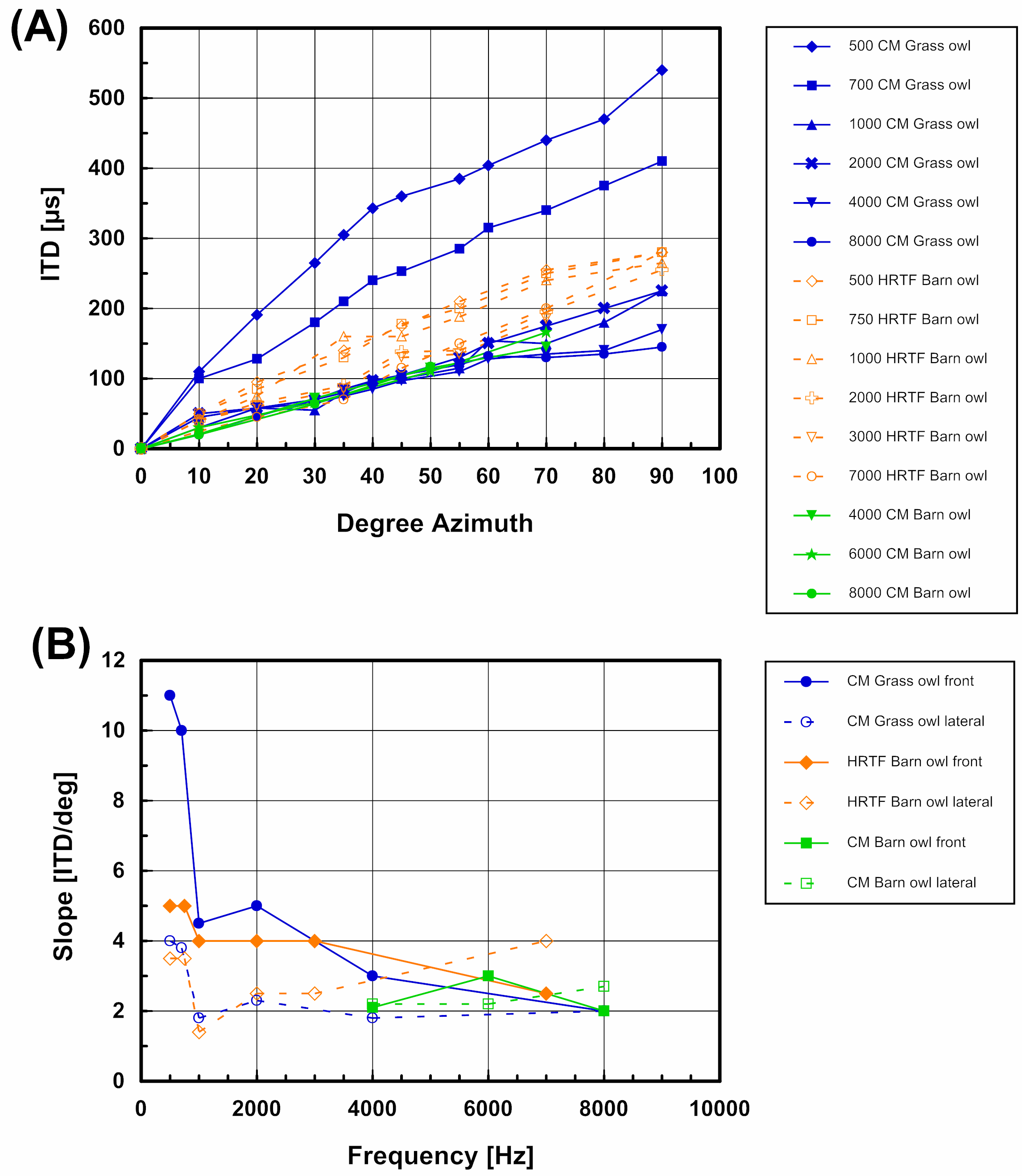

Supplement: S2 Fig — (A) Dependence of ITD was calculated from cochlear microphonics (CM; closed symbols and solid lines) and head-related transfer functions (HRTF; open symbols and dashed lines). Data are estimated from Moiseff 1989 [11] (CM Barn owl, N = 1), from Calford and Piddington 1988 [55] (CM Grass owl, N = 1), and from Hausmann et al. 2010 [52] (HRTF Barn owl, N = 1). HRTF data represent the passive acoustic case, and CM data represent the internally coupled ears case. (B) From the data depicted in (A) we calculated the slopes of the ITD representation. Slopes representing frontal space within ±10° in azimuth are represented by closed symbols and solid lines, whereas slopes representing lateral space are represented by open symbols and dashed lines. The calculation of the lateral slopes are based on data points that approximately correspond to our lateral reference positions of ±45°. For the CM Grass owl data and the HRTF Barn owl data we considered data points within ±35° to ±55° in azimuth, and for the CM Barn owl data we could only consider data points within ±30° to ±50° in azimuth. (TIF) [file pone.0220652.s002.tif]
